# TiO_2_ and N-TiO_2_ Sepiolite and Zeolite Composites for Photocatalytic Removal of Ofloxacin from Polluted Water

**DOI:** 10.3390/ma13030537

**Published:** 2020-01-23

**Authors:** Michela Sturini, Federica Maraschi, Alice Cantalupi, Luca Pretali, Stefania Nicolis, Daniele Dondi, Antonella Profumo, Valentina Caratto, Elisa Sanguineti, Maurizio Ferretti, Angelo Albini

**Affiliations:** 1 Department of Chemistry, University of Pavia, via Taramelli 12, 27100 Pavia, Italy; federica.maraschi@unipv.it (F.M.); alice.cantalupi01@universitadipavia.it (A.C.); luca.pretali@gmail.com (L.P.); stefania.nicolis@unipv.it (S.N.); daniele.dondi@unipv.it (D.D.); antonella.profumo@unipv.it (A.P.); angelo.albini@unipv.it (A.A.); 2 Department of Chemistry and Industrial Chemistry, University of Genoa, via Dodecaneso 31, 16146 Genova, Italy; carattovalentina@gmail.com (V.C.); elisa.sanguineti@gmail.com (E.S.); ferretti@chimica.unige.it (M.F.)

**Keywords:** sepiolite, zeolite, photocatalysis, TiO_2_-based composites, fluoroquinolone, water remediation

## Abstract

TiO_2_ sepiolite and zeolite composites, as well the corresponding N-doped composites, synthesized through a sol–gel method, were tested for the photocatalytic degradation of a widespread fluoroquinolone antibiotic (ofloxacin) under environmental conditions. The catalysts were characterized by X-ray diffraction (XRD), Brunauer–Emmett–Teller (BET), scanning electron microscopy (SEM), and diffuse reflectance spectroscopy (DRS) analyses. A complete drug degradation occurred in 10–15 min in the presence of both TiO_2_ sepiolite and zeolite catalysts, and in 20–30 min with the N-doped ones. Sepiolite proved to be a better TiO_2_ support compared to the most common zeolite both in terms of adsorption capacity and photocatalytic efficiency in pollutants degradation. The influence of nitrogen doping (red shift from 3.2 to 3.0 eV) was also investigated. Although it was blurred by a marked increase of the particle dimension and thus a decrease of the specific surface area of the doped catalysts, it allowed a faster drug removal than direct photolysis. The photochemical paths and photoproducts were investigated, too.

## 1. Introduction

Water quality and water re-use are two emerging issues related to the most important natural resource on which the entire life of the planet depends. To date, it has been established that the conventional wastewater treatment plants are not suitable for the quantitative abatement of a wide range of chemicals, the so-called Contaminants of Emerging Concern (CEC), which are re-entering the bodies of water, even though at very low concentration levels. The removal of CEC, especially pharmaceuticals and personal care products (PPCPs) from waters is an urgent task. These have been detected worldwide in surface waters, drinking waters, wastewater effluents, and soils. Although some drugs have been recently integrated in the list of priority of pollutants by water legislation [1,2,3,4], most of them are not regularly monitored, and the potentially negative environmental implications due to long-time exposure at trace levels cannot be ignored, as recently demonstrated by many studies [4,5]. In particular, antibiotics pose a serious threat because they contribute to the emergence of antibiotic-resistant bacterial strains. In order to provide safe water and to send the reclaimed water back to the water bodies, any new propositions that are sustainable and cost-effective are welcome [4].

Among advanced oxidation processes (AOPs), heterogeneous photocatalysis is considered one of the most promising methods for water decontamination. Various semiconductors can be used as photocatalysts, but TiO_2_ is the most commonly used one due to its well-known advantages, such as chemical stability, non-toxicity, low cost, and photocatalytic efficiency. In recent years, TiO_2_ powder and TiO_2_ composite materials were extensively applied for the removal of contaminants of emerging concern from waters and wastewaters, especially pharmaceuticals and personal care products [6,7,8,9]. As is well documented in the literature, TiO_2_ leads to the degradation and complete mineralization of organics and of their photoproducts, which are often not specifically investigated, although they may preserve considerable toxic effects [8,10]. Despite its effectiveness, TiO_2_ suffers from two important drawbacks if it has to be seriously considered for a large-scale ‘green’ application: the low density of the solar flux and its limitation to the UV light range on the one hand, and its poor employment for bulk industrial use on the other. In the following, we report some of our recent results aiming to improve the applicability of TiO_2_ through modification, which are intended both to extend its sensitivity toward solar light irradiation and to promote the mass transfer minimizing the charge recombination. Doping with non-metal elements, such as nitrogen for example, is considered an efficient and well-established strategy that is meant to enhance the visible light activity of TiO_2_ by reducing the large band gap of this catalyst [11]. On the other hand, the use of different supports increases the active surface area, favoring the interactions between pollutants and the catalyst [11]. Raw materials, such as clay minerals, zeolites, and mesoporous and microporous materials are particularly suitable to this purpose, because they may combine the photocatalytic activity of TiO_2_ with the adsorbent capacity of the support. In particular, zeolites and sepiolites were chosen for different reasons: (i) in view of their morphological and structural properties (i.e., large surface area, high porosity, microporous and fibrous morphology [12,13], (ii) because they have the additional advantage of being green and safe materials mostly available in nature, and (iii) they showed a great affinity toward fluoroquinolones (FQs) [14,15].

To date, various TiO_2_–zeolite composites have been prepared and used for the degradation of pharmaceuticals [14,16,17,18,19,20,21,22,23,24,25,26,27,28], whereas sepiolites have not been studied toward this aim.

In the present paper, the effectiveness of N-doped and undoped sepiolite and zeolite TiO_2_ supports was compared for the removal of a largely used human medicine, the antibiotic ofloxacin (OFL). This molecule was chosen as a representative antimicrobial for a range of reasons, viz. (i) it is largely employed for human diseases and frequently detected in environmental waters, (ii) a certain antimicrobial activity is maintained even at advanced decomposition due to the strong stability of the quinolone ring that slows down the degradation rate of these thermodynamically resistant drugs, (iii) their strong UV-A absorption makes such drugs ideal for quantitative studies, and (iv) its photochemistry was previously investigated under environmental conditions by our group [29,30,31]. Thus, the study was carried out in tap water and under simulated sunlight. The photodegradation profiles of OFL and its photoproducts were studied, and the photoproducts’ structures were identified. The resulting sludge was compared with those obtained in the presence of a suspension of TiO_2_.

## 2. Materials and Methods

### 2.1. Materials

All the chemicals employed were reagent grade or higher in quality. OFL, zeolite Y in sodium form, sepiolite powder, titanium (IV) tetraisopropoxide (97% v/v), and 2-propanol (99.5% v/v) were purchased from Sigma–Aldrich (Milano, Italy). High Performance Liquid Chromatography (HPLC) gradient grade acetonitrile (ACN) was purchased by VWR International (Milano, Italy), H_3_PO_4_ (85% w/w) and ammonia solution (30% w/w) were purchased by Carlo Erba Reagents (Cornaredo, Milano, Italy). Aqueous OFL solutions were prepared and stored in the dark at 4 °C before use.

### 2.2. Preparation and Morphostructural Characterization of the Composite Catalysts

Each catalyst was synthesized through a solid-state procedure as described below and in Table 1, and characterized by means of XRD, BET, SEM, and DRS analyses.

#### 2.2.1. Zeolite–TiO_2_ Synthesis via Sol–Gel Route

In order to synthesize TiO_2_, titanium tetraisopropoxide, isopropanol, and water (1:2:10) were mixed together for 4 h under vigorous magnetic stirring; then, the gel was thermally treated at 100 °C for 12 h. Eventually, amorphous TiO_2_ is obtained (100 mg mL^−1^) as a white powder, and afterwards, it was mixed to 50 mg mL^−1^ zeolite in deionized water. The suspension was kept under magnetic stirring for 20 min, filtered, washed, and dried in an oven for one night at 100 °C. At the end, the samples were thermally treated in a muffle at 350 °C for 1 h in order to obtain the crystallization of TiO_2_ from the amorphous to the anatase phase (ZT) [14]. N-doped TiO_2_ supported on zeolite (ZTN) was synthetized as above described, using 15% ammonia solution as solvent [32].

#### 2.2.2. Sepiolite–TiO_2_ Synthesis via Sol–Gel Route

Two different methods were used to synthesize sepiolite–TiO_2_ samples at different ratios. The first procedure is similar to the one used for the zeolite–TiO_2_ synthesis: 10 mg mL^−1^ of commercial sepiolite were added to an aqueous solution of amorphous TiO_2_ (250 mg mL^−1^), and the suspension was stirred for 30 min. Then, the samples were centrifuged at 2500 rpm for 10 min and dried in an oven at 100 °C for one night. The powder was calcined at 500 °C for 1 h, washed with deionized water, centrifuged three times in order to remove not bound sepiolite, and eventually dried in an oven for one night at 100 °C (ST-1).

The second procedure involves the addition of sepiolite during the sol–gel synthesis: 2 g of sepiolite were added to titanium tetraisopropoxide, isopropanol, and water (1:2:10) and left under magnetic stirring for 4 h. The samples were dried in an oven for one night at 100 °C (ST-2). Afterwards, the powder was calcined at 500 °C for 1 h, washed with deionized water, centrifuged three times, and eventually dried in an oven for one night. N-doped TiO_2_ supported on sepiolite (STN-1 and STN-2) was synthesized as above described, using 15% ammonia solution as solvent [32].

#### 2.2.3. Morphostructural Characterization

Phase identification was performed by X-ray powder diffraction analysis using a Philips PW1830 diffractometer (Philips, Amsterdam, The Netherlands) using Bragg–Brentano geometry; Cu Kα; Ni filtered; 2θ range 20–80°; step 0.025°; sampling time 10 s. Brunauer–Emmett–Teller (BET) analysis was carried out using an ASAP 2010 physisorption analyzer (Micromeritics Instrument Corp., Norcross, GA, USA). Samples were pre-treated at 200 °C in vacuum, before analysis.

Microstructural characterization was performed with a high-resolution scanning electron microscope (SEM, TESCAN Mira 3 XMU, TESCAN, Brno, Czech Republic) operated at 25 kV. The composition microanalysis was determined by energy-dispersive spectroscopy (EDS, EDAX Inc., Mahwah, NJ, USA). The samples were previously coated with carbon using a Cressington coater HR 208. DRS spectra were acquired in the wavelength range of 300–800 nm by using a 145 Jasco V-750 spectrophotometer (Jasco Europe, Lecco, Italy) equipped with an integrating sphere (Jasco ISV-922, Jasco Europe, Lecco, Italy). Quartz 146 cuvettes (VWR International, Milano, Italy) with 1 mm optical path were used.

### 2.3. Adsorption Experiments

OFL adsorption onto the six systems was studied using a batch equilibration method. OFL solutions were prepared in tap water at different initial concentrations (*C_0_*) ranging from 3 to 300 mg L^−1^. First, 25 mg of each catalyst was weighed into polypropylene centrifuge tubes and mixed with 50 mL of FQ solutions. The tubes, wrapped with aluminum foils to prevent FQ light-induced decomposition, were shaken at 150 rpm for 24 h at room temperature (20 ± 1 °C) in order to ensure equilibration. The suspensions were filtered through a 0.22 µm membrane filter for analysis. The OFL concentrations at equilibrium (*C_w_*) in the filtered solutions were measured by a UV-Vis spectrophotometer. The OFL adsorbed amounts (*C_s_*) were calculated from the difference between C_0_ and C_w_. All experiments were performed in triplicate with good reproducibility (RSD <10%), while control solutions of OFL with no catalyst were also measured. No changes in FQ concentrations were detected in the control samples.

### 2.4. Irradiation Experiments

Irradiation was performed by using a solar simulator (Solar box 1500e, CO.FO.ME.GRA, Milano, Italy) set at a power factor of 500 W m^−2^, equipped with a UV outdoor filter of soda lime glass, and IR treated. First, a 100 mL tap water sample from the municipal waterworks of Pavia (pH 7.7, conductivity at 20 °C 271 μS cm^−1^, Ca^2+^ 35 mg L^−1^, Mg^2+^ 10 mg L^−1^, Cl^−^ 5 mg L^−1^, NO_3_^−^ 0.6 mg L^−1^, SO_4_^2−^ 5 mg L^−1^) spiked with 10 mg L^−1^ OFL was irradiated in a closed glass container (40 mm depth, exposed surface 9500 mm^2^).

Catalyst suspensions (0.010 g L^−1^ OFL, 0.5 g L^−1^ TiO_2_) were magnetically stirred in the dark for 20 min to promote the antibiotic adsorption on the catalyst surface. During the irradiation course, aliquots (ca. 1 mL) of each sample (100 mL) were withdrawn at specific times and measured by HPLC-UV after filtration (0.22 µm).

For the photoproducts’ identification, 100 mL samples of a 0.020 g L^−1^ OFL solution containing 0.5 g L^−1^ of the catalyst were magnetically stirred in the dark for 20 min and then irradiated in a closed glass vessel under continuous magnetic stirring. The irradiated samples were immediately filtered (0.22 µm) and analyzed by HPLC-UV (Shimadzu Corporation, Milano, Italy) and HPLC-ESI-MS/MS (Thermo Finnigan, San Josè, CA, USA). 

### 2.5. Analytical Determinations

A UVmini-1240 UV-Vis spectrophotometer (Shimadzu Corporation, Milano, Italy) fixed at 288 nm was used for the absorption experiments. Calibrations with four standards at concentrations between 1 and 10 mg L^−1^ yielded optimal linearity (R^2^ >0.9996). 

The HPLC-UV system consisted of a Shimadzu LC- 20AT solvent delivery module (Shimadzu Corporation, Milano, Italy) equipped with a DGU-20A3 degasser (Shimadzu Corporation, Milano, Italy) and interfaced with a SPD-20A UV detector (Shimadzu Corporation, Milano, Italy). The wavelength selected for analysis was 275 nm. Then, 20 µL of each sample was injected into a 250 × 4.6 mm, 5 µm Analytical Ascentis C18 (Sigma-Aldrich, Milano, Italy) column coupled with a similar guard column. The mobile phase was 25 mM H_3_PO_4_-ACN (85:15) at a flow rate of 1 mL min^−1^. The instrumental quantification limit was 0.06 mg L^−1^.

The HPLC–ESI-MS/MS (Thermo Finnigan, San Josè, CA, USA) analyses for the photoproducts’ identification were performed by using a Thermo-Finnigan LCQ ADV MAX ion trap mass spectrometer equipped with an ESI ion source and a Surveyor HPLC system equipped with a Phenomenex Jupiter 4u Proteo (150 × 2.0 mm, 4 μm) column. ESI experiments were carried out in positive ion mode under the following constant instrumental conditions: source voltage 5.0 kV, capillary voltage 46 V, capillary temperature 210 °C, tube lens voltage 55 V. The elution was performed using 0.1% HCOOH in ultrapure water (solvent A) and 0.1% HCOOH in ACN (solvent B) at a flow rate of 0.2 mL min^−1^, and the injection volume was 100 µL. Elution started with 98% solvent A for 5 min followed by a linear gradient from 98% to 0% A in 40 min, 0% A for 5 min, and 98% A for 10 min. MS/MS spectra obtained by collision-induced dissociation were performed with an isolation width of 2 Th (m/z), and the activation amplitude was around 35% of the ejection radio-frequency amplitude of the instrument.

## 3. Results and Discussion

In the present work, different TiO_2_ sepiolite and zeolite composites, as well the corresponding N-doped TiO_2_ composites, were critically compared under environmental conditions for the photocatalytic degradation of the antibiotic OFL, which was chosen as a more suitable probe with respect to the commonly used dyes, as there may be a confounding issue in view of their sensitization under irradiation [33].

### 3.1. Morphostructural Characterization 

The synthesized composites were characterized by means of XRD, BET, SEM, and DRS analyses. 

**Figure 1:**
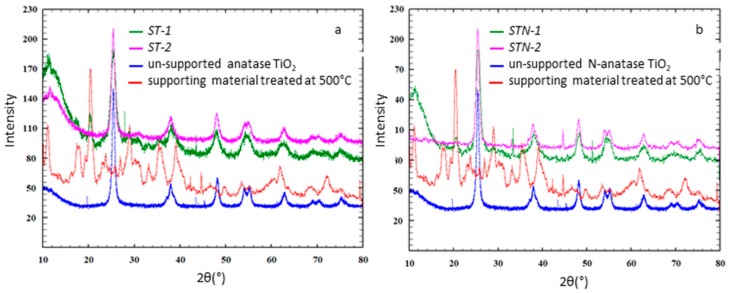
XRD spectra of samples (**a**) ST-1 (green), ST-2 (pink), unsupported anatase TiO_2_ (blue), the supporting material treated at 500 °C (red) and (**b**) STN-1 (green), STN-2 (pink), unsupported N-anatase TiO_2_ (blue), the supporting material treated at 500 °C (red).

As apparent in XRD patterns (Figure 1), all the composites have the anatase structure, as also pointed out by the reference pattern [14]. 

BET analysis identified the specific surface area values among the synthesized samples, as shown in Table 2.

The highest value was obtained for ST-1, which is equal to 262 m^2^ g^−1^, while the lowest value was obtained for ZTN, equal to 108 m^2^ g^−1^. The other samples had intermediate values: 159, 228, 166 and 153 m^2^ g^−1^ for ST-2, ZT, STN-1 and STN-2, respectively.

**Figure 2:**
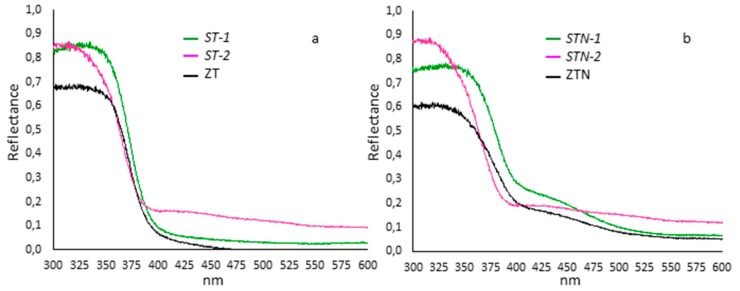
DRS spectra of (**a**) ST-1 (green), ST-2 (pink), and ZT (black) and (**b**) STN-1 (green), STN-2 (pink) and ZTN (black).

The E_gap_ values of the semiconductor synthetized samples were derived by using the wavelength corresponding to the edge of the experimental spectrum and the relation: ΔE_gap_ = c/k (Kubelka–Munk equation). The DRS spectra of N-doped samples reveal the presence of a red shift absorption edge with an absorption tail extending in the visible region originated from the new energy levels in the forbidden band of TiO_2_ formed by N-doping (see Figure 2) [34,35]. As expected, the undoped samples, ST-1 and ZT showed a band gap close to 3.2 eV, while the N-doped samples, STN-1 and ZTN had a smaller band gap, close to 3.0 eV, confirming the E_gap_ values of nitrogen-doped and undoped TiO_2_ nanoparticles [32]. Only the ST-2 and STN-2 samples exhibited similar spectra corresponding to a band gap of 3.20 and 3.18 eV, respectively. 

### 3.2. Adsorption Isotherms

**Figure 3:**
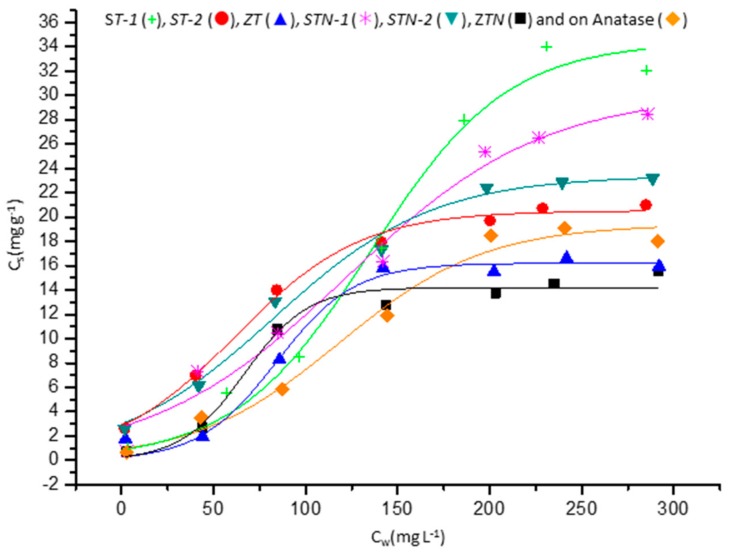
Adsorption profiles of OFL onto the six systems.

The literature reports different models to fit the experimental data from adsorption isotherms [36]. As shown in Figure 3, the experimental adsorption profiles of OFL both onto the composite materials and on TiO_2_ anatase display a sigmoidal trend, which usually occurs in the presence of a cooperative adsorption. This indicates that at lower OFL concentration in the solution, the adsorption is low, while it increases when the solute concentration increases. This behavior has been already observed in previous studies regarding the adsorption of organic molecules onto clay minerals and sepiolites [15,36,37,38].

(1) $$C_{s}=\frac{C_{s\max}}{1+e^{-A(C_{w}-F)}}$$

Equation (1) describes the sigmoidal curve. *C*_s max_ is the maximum amount of molecules adsorbed, *A* is a coefficient indicating the efficiency of the adsorption mechanism, and *F* represents the inflection point.

The isothermal parameters of the model were obtained by the non-linear fitting performed with dedicated software (Origin^®^) and are listed in Table 3. The good correlation coefficients (R^2^) shows that the sigmoidal shape isotherm (Equation (1)) fits the experimental data accurately. 

As apparent in Figure 3, TiO_2_ zeolites cause only a small effect on the adsorption (although the maximum value is reached earlier), while TiO_2_ sepiolites may reach a value that is twice as large.

### 3.3. Photodegradation Kinetics

The photocatalytic effectiveness of the six systems (ST-1, ST-2, ZT, STN-1, STN-2, and ZTN) was tested against OFL in tap water under simulated solar light. Tap water was chosen for its invariant composition and greater similarity to surface water than ultrapure water; moreover, the OFL degradation rate constant is not different from that obtained in environmental waters [30].

Before irradiation, spiked samples (0.01 g L^−1^ OFL, 0.5 g catalyst) were stirred in the dark for 20 min, which was the amount of time required to ensure FQ absorption onto the catalyst surface and to achieve equilibrium between an FQ adsorbed fraction and FQ fraction in solution. Different amounts of OFL were adsorbed on each catalyst (see Table 4). 

**Figure 4:**
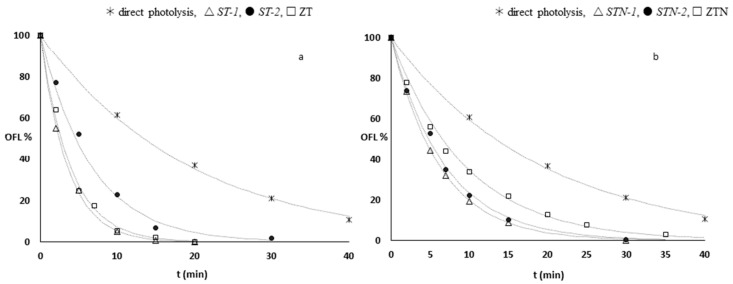
Kinetic profiles of the antibiotic under direct photolysis and in presence of (**a**) undoped and (**b**) doped TiO_2_-supported catalysts.

The degradation profiles of the antibiotic under direct photolysis and doped and undoped TiO_2_-supported photocatalysis are shown in Figure 4.

The degradation rate (*r*) of OFL, expressed by the Equation (2)

(2) $$r=k_{deg}\times C_{w},$$

was greatly enhanced by the presence of all the additives. After 10 min of irradiation with no catalyst, more than 50% of OFL was still present, while the abatement was quantitative (≥95%) in the presence of undoped composites, ST-1 and ZT. Lower degradation rates were observed in the presence of ST-2 and of all the doped catalysts. In particular, a quantitative abatement was achieved in 20 min in the presence of both TiO_2_ N-doped sepiolite catalysts, while 30 min was required with ZTN.

All experimental data were fitted by a monoexponential first-order law (Equation (3)), using dedicated software (Fig.P application, Fig.P Corporation Software, version 2.2a, BIOSOFT, Cambridge, UK):

(3) $$\mathrm{OFL}\%=100\;\times e^{-k_{deg}\;t}.$$

**Figure 5:**
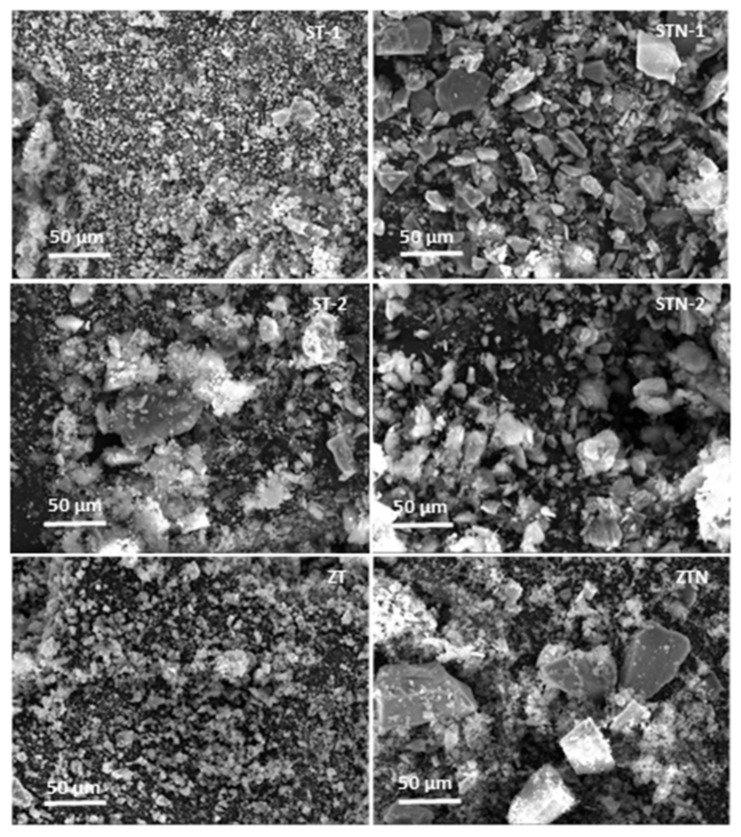
SEM of the six systems.

**Figure 6:**
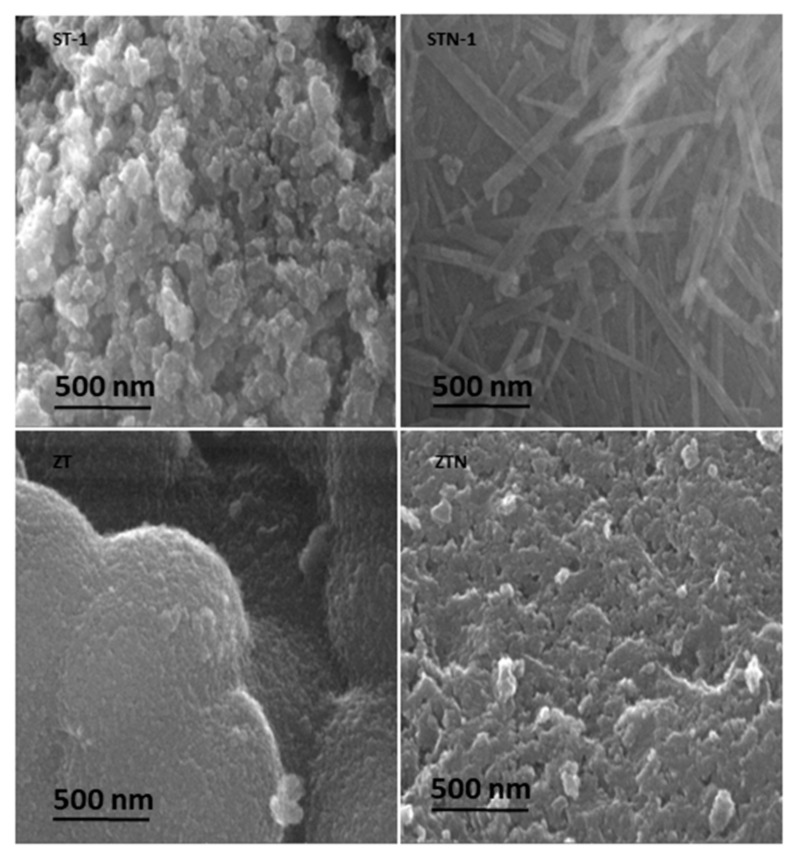
Nanostructure (150 kx) of ST-1, STN-1, ZT, and ZTN.

In Table 4, the kinetic degradation constants (*k_deg_*), expressed in min^−1^ are reported. As observed, the kinetic degradation constants values obtained in the presence of all the N-doped materials appear to be about 40–60% lower than those obtained with TiO_2_ undoped composites. This behavior may be ascribed to a strong decrease of the specific surface area due to the doping treatment, resulting in a net decrease of the photocatalytic efficiency. It has been demonstrated in the literature that the presence of nitrogen as a dopant modifies the nanoparticles’ morphology [32,39,40,41]. SEM images (Figure 5 and Figure 6) confirm this behavior. Specifically, undoped catalysts, ST-1 and ZT, are composed of spherical nanoparticles that form small, soft, and porous agglomerates of a few micrometers characterized by a large surface area. On the contrary, all nitrogen-doped composites show bigger, irregular aggregates up to 50 µm that clearly affect their photocatalytic activity. Moreover, thin lamellar nanostructures growing toward a preferential direction (c axis) are observed especially in STN-1. ST-2 and STN-2 are less homogeneous materials with a high level of agglomeration. The TiO_2_ distribution on ST-2 and STN-2 was verified by EDS mapping (Figure S1). 

In order to distinguish the contribution of the band-gap variations among the photocatalysts studied, a superposition of the emitted light and the absorption spectrum gave information on the effective light absorbed, which is 6% and 4% for ST-1 and ZT, and 11% and 8% for STN-1 and ZTN, respectively. Both ST-2 and STN-2 absorption is around 12–14%. As shown in Figure 2, the band gaps are on the near-UV range, where the simulated solar light gives a small contribution, resulting in a scarce contribution to the photoreactivity. On the other hand, N-doping markedly shifts the light absorption edge of TiO_2_ toward visible light and still allows for a quick removal of OFL as compared to direct photolysis.

The ratio sepiolite/TiO_2_ employed for the syntheses of ST-2 and STN-2 catalysts seems to have a negligible influence on the degradation rate constants and on the band gap shift both of doped and undoped catalysts, as shown in Table 2 and Figure 2. Although the synthesis procedure is a shorter thermal treatment than the one reported in the literature [42], ST-2 and STN-2 are not as effective as ST-1 and STN-1 in the OFL removal. 

In conclusion, such materials require few simple repeatable synthetic steps and may be more palatable for bulk industrial use, since they favor both catalyst recovery and allow activating the photocatalytic degradation of emerging pollutants using solar light.

### 3.4. Photoproducts Identification

A larger number of intermediates was identified in the present study. The maximal amount of primary intermediates during the course of the oxidation never exceeded 10% with respect to the initial amount of the parent compound, and it was reached after a few minutes of irradiation. The lifetimes of all the photoproducts were of the same order of that of the starting OFL, and in some cases they were even shorter. 

HPLC-ESI-MS/MS analysis showed a marked difference in the photoproducts distribution between catalyzed, either unsupported TiO_2_, ZT, or ST series catalysts (see Table S1), and uncatalyzed OFL degradation [43]. In the latter case, degradation proceeds through the triplet excited state of the parent molecule and three photoproducts families can be evidenced, arising from three different competitive reaction pathways, viz. electron-rich side-chains oxidation, reductive de-halogenation, and fluorine substitution by a hydroxyl group [43]. On the contrary, when the reaction is carried out in the presence of a photocatalyst, the side chain oxidative degradation becomes by far the predominant path. The vast majority of the products come via hole transfer from the excited catalyst to the adsorbed OFL, and they are either followed by a cascade reaction leading to a plethora of oxidized products mainly affecting the electron rich side-chains, viz. a hydroxylation-initiated cleavage of the piperazine and morpholine rings, or leading to the substitution of a hydroxyl for the 6-fluoro group (O10, 013, 019). Both types of reactions have been found to occur upon treatment of FQs by a monoelectronic oxidant such as the sulfate radical [44]. Only a few products of reductive dehalogenation (O6, O7, O9, O21, O27, O31, and O32) have been characterized, and these can be ascribed to an energy transfer between titania nanoparticles and OFL producing the triplet of the drug (ET <2.7 eV) [43].

Some of the photoproducts have already been identified and characterized in our previous works [30,45,46], the remaining structures proposed via HPLC-ESI-MS/MS analysis and relevant fragmentations are described in the Supplementary Material. Thus, it appears from the identified degradation products that both ZT and ST photocatalysts maintain the same reaction mechanism of the original TiO_2_ photocatalyst in OFL degradation, starting an oxidative process that eventually leads to the complete removal of the parent drug from the water, whilst adding the adsorption proprieties of high active surface clays. Furthermore, the shift in the band gap due to N-doping, although reducing quantum efficiency, allows for the use of solar light, eliminating the need of artificial UV lamps.

## 4. Conclusions

In this paper, we report a critical comparison among various TiO_2_ and N-doped TiO_2_ sepiolite and zeolite composites for the photocatalytic degradation of one of the most used human antibiotics. These materials were obtained by a well-established sol–gel synthesis and demonstrated the ability to degrade a suitable model compound with respect to direct photolysis. On the other hand, doped composites were demonstrated to be an inefficient choice, as the red shift of the absorption occurs at the expense of the overall efficiency, since doping necessarily involves a large decrease in specific surface area. This appears to be a general property among N-doped catalysts. The photocatalytic efficiency was studied under environmental conditions, natural water and sunlight; the photochemical paths and photoproducts were investigated, too. The results indicated that sepiolite is a valid TiO_2_ support because it ensures both an exhaustive adsorption and a quick degradation of OFL, thus reaching the most effective employment of titania and a further most expeditious recovery of the catalyst with respect to the nanoparticle TiO_2_. Moreover, in the first steps of the process, the primary photoproducts were accumulated to a sufficient concentration to be identified. Indeed, all of them conserve the highly stabilized quinolone moiety and follow the degradation, with lifetimes similar to that of the starting compound. 

## Figures and Tables

**Table 1:** Experimental conditions for the syntheses of the six systems. ST: TiO_2_ supported on sepiolite, ZT: TiO_2_ supported on zeolite, STN: N-doped TiO_2_ supported on sepiolite, ZTN: N-doped TiO_2_ supported on zeolite. For full descriptions of the short forms, see Section 2.2.1 and Section 2.2.2.

Photocatalysts	Type of Support	Crystallization Temperature (°C)	Treatment Time (h)
**ST-1**	Sepiolite	500	1
**STN-1** ^1^	Sepiolite	500	1
**ST-2** ^2^	Sepiolite	500	1
**STN-2** ^1,2^	Sepiolite	500	1
**ZT**	Zeolite	350	1
**ZTN** ^1^	Zeolite	350	1
**TiO_2_ Anatase**	—	350	1

^1^ 15% ammonia solution as solvent; ^2^ one step synthesis.

**Table 2:** Brunauer–Emmett–Teller (BET) and band gap of the six systems compared to those of TiO_2_ anatase.

Photocatalysts	BET (m^2^ g^−1^)	Band Gap (eV)
ST-1	262	3.18
STN-1	166	3.03
ST-2	159	3.20
STN-2	153	3.18
ZT	228	3.16
ZTN	108	3.03
TiO_2_ Anatase	120	3.21

**Table 3:** Parameters obtained by fitting the experimental data of ofloxacin (OFL) adsorption onto the six systems and on TiO_2_ anatase (in brackets the standard deviations values on the last significant digit).

Photocatalysts	*C_s_*_max_ (mg g^−1^)	F (mg L^−1^)	A	R^2^
ST-1	34 (2)	135 (8)	0.027 (4)	0.98
STN-1	30 (2)	122 (14)	0.019 (3)	0.97
ST-2	20 (1)	62 (3)	0.029 (3)	0.99
STN-2	23 (1)	82 (6)	0.023 (3)	0.98
ZT	16 (1)	84 (4)	0.05 (1)	0.98
ZTN	14 (1)	66 (5)	0.06 (1)	0.97
TiO_2_ Anatase	19 (1)	118 (9)	0.025 (4)	0.98

**Table 4:** Percentage of OFL adsorbed in the dark for 20 min stirring, kinetic degradation constant values of the six systems compared to those of TiO_2_ anatase (in brackets, the standard deviations values on the last significant digit).

Photocatalysts	% OFL (20 min, Dark)	*k_deg_* (min^−1^)
ST-1	17	0.289 (1)
STN-1	22	0.162 (2)
ST-2	43	0.145 (6)
STN-2	43	0.131 (8)
ZT	25	0.26 (1)
ZTN	31	0.106 (3)
TiO_2_ Anatase	20	0.31 (1)

## Supplementary Materials

The following are available online at https://www.mdpi.com/1996-1944/13/3/537/s1, Table S1: Fragmentation of ZT and ST-photocatalytic products of OFL. The letters in brackets indicates the reaction conditions where that product has been identified, viz. in the presence of TiO_2_ Zeolite (ZT) composite, TiO_2_ Sepiolite composite (ST) or unsupported TiO_2_ (OFL), Figure S1: EDS mapping (4.00 kx) of Si and Ti in ST-2 showing the TiO_2_ distribution on the surface of the catalyst: BSE (back-scattered electrons) image of ST-2 (a), Si distribution (b), Ti distribution (c).
