# Supplementary material for: TiO2 and N-TiO2 Sepiolite and Zeolite Composites for Photocatalytic Removal of Ofloxacin from Polluted Water
Source: Materials (Basel). 2020 Jan 23;13(3):537. doi: 10.3390/ma13030537 (PMC7040821; doi:10.3390/ma13030537)
Supplement: Supplementary file 1 [file materials-13-00537-s001.pdf]

materials

Supplementary

TiO2 and N-TiO2 Sepiolite and Zeolite Composites for Photocatalytic Removal of Ofloxacin from Polluted Water

Michela Sturini [1,*], Federica Maraschi [1], Alice Cantalupi [1], Luca Pretali [1], Stefania Nicolis [1], Daniele Dondi [1], Antonella Profumo [1], Valentina Caratto [2], Elisa Sanguineti [2], Maurizio Ferretti [2] and Angelo Albini [1]

[1] Department of Chemistry, University of Pavia, via Taramelli 12, Pavia 27100, Italy; federica.maraschi@unipv.it (F.M.); alice.cantalupi01@universitadipavia.it (A.C.); luca.pretali@gmail.com (L.P.); stefania.nicolis@unipv.it (S.N.); daniele.dondi@unipv.it (D.D.); antonella.profumo@unipv.it (A.P.); angelo.albini@unipv.it (A.A.)

[2] Department of Chemistry and Industrial Chemistry, University of Genoa, via Dodecaneso 31, Genova 16146, Italy; carattovalentina@gmail.com (V.C.); elisa.sanguineti@gmail.com (E.S.); ferretti@chimica.unige.it (M.F.)

* Correspondence: michela.sturini@unipv.it; Tel.: +39-0382-987347

Table S1. Fragmentation of *ZT* and *ST*-photocatalytic products of OFL. The letters in brackets indicates the reaction conditions where that product has been identified, viz. in the presence of TiO_2 Zeolite (ZT) composite, TiO_2 Sepiolite composite (ST) or unsupported TiO_2 (OFL).

ID	Structure	ID	Structure
O1 (*OFL*)	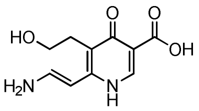	O18 (*ZT, ST*)	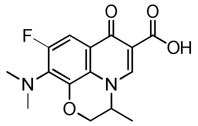
O2 (*OFL, ZT*)	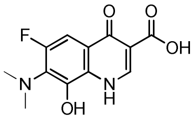	O19 (*ZT, ST*)	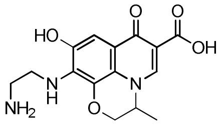
O3 (*OFL, ZT,ST*)	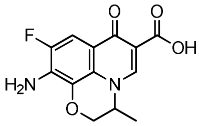	O20 (*ZT, ST*)	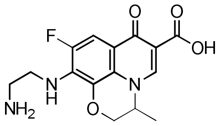
O4 (*OFL, ZT, ST*)	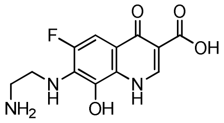	O21 (*ZT, ST*)	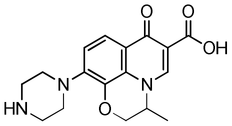
O5 (*OFL*)	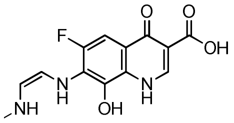	O22 (*ZT*)	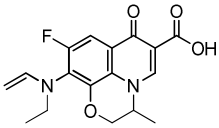

O6 (*OFL*)

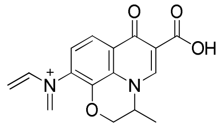

O23 (*ZT, ST*)

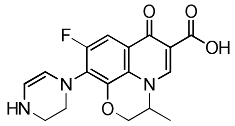

O7 (*OFL*, *ZT*, *ST*)

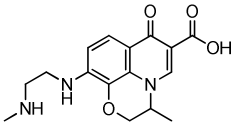

O24 (*ZT, ST*)

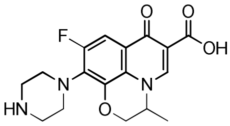

O8 (*OFL, ST*)

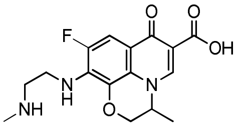

O25 (*ZT*)

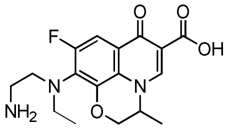

O9 (*OFL, ST*)

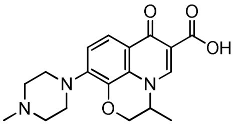

O26 (*ZT, ST*)

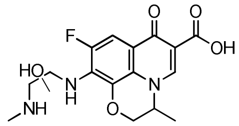

O10 (*OFL*)

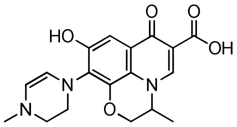

O27 (*ZT*)

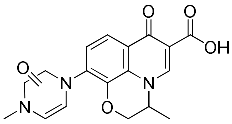

O11 (*OFL, ZT, ST*)

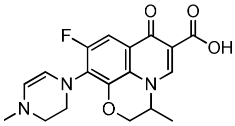

O28 (*ZT, ST*)

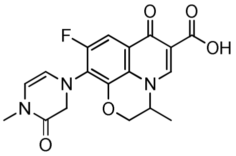

O12 (*OFL, ZT*)

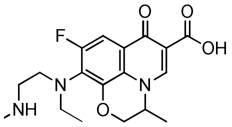

O29 (*ZT*)

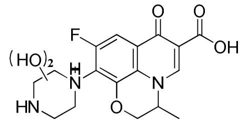

O13 (*OFL, ZT, ST*)

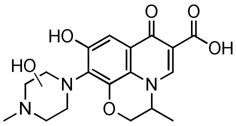

O30 (*ZT*)

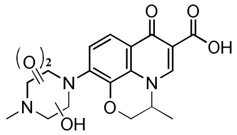

O14 (*OFL, ZT, ST*)

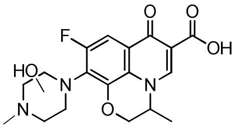

O31 (*ST*)

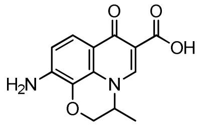

O15 (*OFL*)

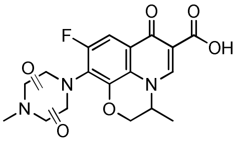

O32 (*ST*)

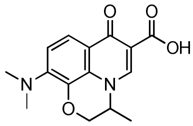

O16 (*OFL, ZT*)

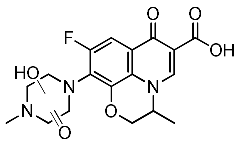

O33 (*ST*)

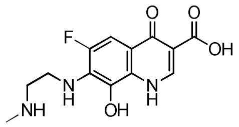

O17 (*ZT, ST*)

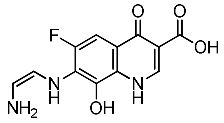

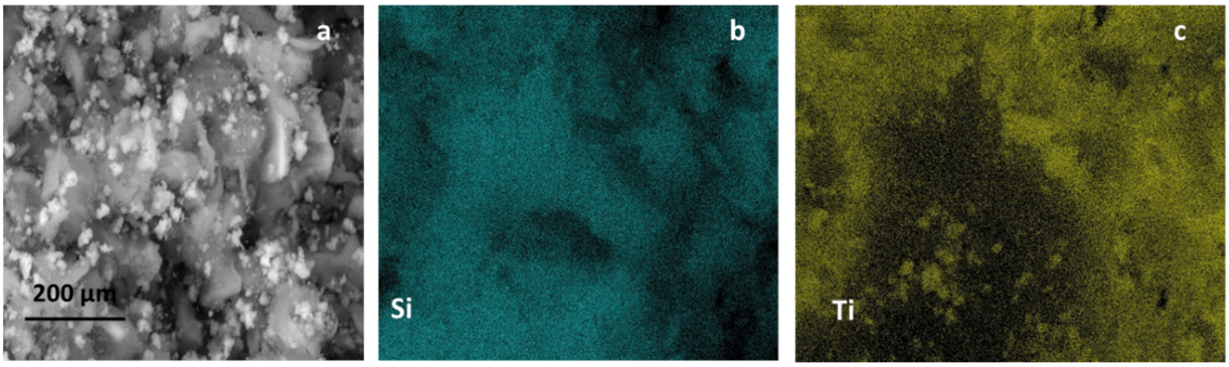

Figure S1. EDS mapping (4.00 kx) of Si and Ti in *ST-2* showing the TiO_2 distribution on the surface of the catalyst: BSE (back-scattered electrons) image of *ST-2* (**a**), Si distribution (**b**), Ti distribution (**c**).

© 2020 by the authors. Licensee MDPI, Basel, Switzerland. This article is an open access article distributed under the terms and conditions of the Creative Commons Attribution (CC BY) license (http://creativecommons.org/licenses/by/4.0/).
